# Remarks on the Two Hypotheses Produced by M. Lavoisier, to Account for the Elasticity of Aëriform Fluids, with an Attempt to the Further Illustration of the Subject

**Published:** 1801-06

**Authors:** 


### *Remarks on the two Hypotheses produced by* M. Lavoisier, *to account for the Elasticity of Aëriform Fluids, with an Attempt to the further Illustration of the Subject.*

"Follow Nature."

M. LAVOISIER, in the first chapter of his Elements of Chemistry, treats on the formation of aëriform fluids, and ends the article, by illustrating the cause of their elasticity. He says, that, "It is by no means difficult to perceive that their elasticity depends on that of caloric, which seems to be the most eminently elastic body in nature." Now it would seem that the elasticity of aëriform fluids is not dependent altogether on caloric, merely as partaking in common with its properties; for, upon the consideration of circumstances, and the change which is effected in the arrangement of the particles of a body reduced to the aëriform state, being properly appreciated, its elasticity would appear to be depending more on the increased force given to the action of the repulsive power,* inherent between its particles.

Nothing appears more easy of conception, before we enter upon enquiry, than that one body, by entering into combination, should partake of the quality of another; and thus, because caloric appears to possess the quality of elasticity in a more eminent degree than any other body in nature, it perhaps would appear extremely plausible to account in this way for the elasticity of aëriform fluids, and content ourselves by saying that caloric conveys the quality of elasticity to bodies; but enquiry and consideration make it appear that this is a very undetermined mode of explanation. It cannot answer a question, nor remove a doubt on the subject, and leaves us entirely ignorant what elasticity is. Can we be satisfied that caloric retains the quality of elasticity, after its diffusion through bodies? We are to consider that a combination takes place *chemically*, between the bases of aëriform fluids and caloric; and how far the remaining caloric, in diffusion, retains its elasticity as when in a free state, must also remain doubtful, as long as it is a query, whether caloric does not lose this quality when in a quiescent state? or, at least, whether it does not suffer a partial deprivation of it? for elasticity is a quality wholly dependent on circumstance, and not an inherent quality, which a body

* M. Lavoisier has produced a confusion in our ideas on the power of repulsion, by confounding the effect of caloric with the operations of the repulsive power itself.

a body retains through all its modifications. A body in one ſtate may be non-elaſtic; in another, the moſt highly elaſtic of any body in nature. And as caloric is ſubject to combination, (indeed more ſo than any other body) we have a right to ſuppoſe that it is alſo ſubject to modification. And, further, that it is alſo ſubject to a *total* change of property; which ſuppoſition muſt evince the impropriety of accounting for the elaſticity of aëriform fluids, by ſuppoſing they partake of it with caloric, ſince the poſſibility is evident of its being deprived altogether of this quality upon combination, or even upon a ſlight modification of its exiſtence.*

M. Lavoiſier produces a difficulty in conceiving how the repulſive power acts upon very minute particles, placed at great diſtances from each other; that is, I ſuppoſe, he conſiders all connection loſt, for, while there is connection, there can be no difficulty in the ſuppoſition. It is very natural to conclude that the particles are connected from the preſſure always exerted on bodies expoſed to the atmoſphere, and likewiſe from the attractions exiſting between them, whoſe force of action we may reaſonably compute to be about equal to the contrary one exerted by the repulſions between the particles of compact bodies; for the two powers under theſe circumſtances appear to be very ſimilarly ſituated as to their range of action, and ſince it appears that the particles of the moſt compact bodies do not touch each other, from the repulſions exiſting between them. Have we not as much reaſon to imagine that the particles of a flint do not mutually repel each other, as to ſuppoſe that the particles of a gas do not mutually attract each other?

M. Lavoiſier ſeems likewiſe not to have admitted, or at leaſt has not ſufficiently noticed, the modifications of the attractive power in the ſeveral ſtates of rarefaction in bodies. Speaking of the different ſtates of exiſtence of bodies, in one place he ſays, "that they would almoſt inſtantaneouſly paſs from the ſolid ſtate of aggregation to that of aëriform elaſticity, was it not for the preſſure of the atmoſphere. Now this could not happen, without the power of repulſion ſo far overcoming the

* Here caloric is conſidered as a body, in compliance with M. Lavoiſier's doctrine; in ſhort, it is very difficult to conſider it in any other light; for how a property can cauſe a change upon that on which it entirely depends, is yet to be explained. Properties are wholly paſſive, they never change, but are changed; hardneſs is a property of many bodies, but, when they become ſoft, it cannot be ſaid that the *hardneſs* produced the ſoftneſs, or, *that it went away of its own accord*; but, that ſuch bodies being acted upon by ſome external agent entering into a ſtate of modification, loſe this former property of hardneſs, and take on ſuch of the then exiſting circumſtances.

the attractive one, as to produce that degree of separation between the particles of the body, *sufficiently distant*, to give it the aëriform state. Unless the modified states of this power is noticed, it carries with it a sense of its annihilation. For instance; saying "that ice, when exposed to a temperature above 32°, loses its solid form, owing to its particles no longer being held together by reciprocal attraction," is a language that does not carry with it the idea of the modification of the attractive power between the particles, but a total extinction of it. The particles of water, (when fluid) certainly have not so great an attraction to each other, as when they were in the form of ice, yet they certainly have reciprocal attraction, both in this and the vaporous state, unless we can imagine a regeneration of this power upon the condensation of vapour. So requisite is it, that the increase and diminution of the two powers between the particles of bodies should be strictly kept up, by our ideas being made familiar with the different arrangements they undergo upon the change of circumstance, from *rarefaction* to *condensation*, and the *modified states* of each, that without such we can have no true idea of elasticity, which is wholly dependent on the circumstance of such arrangements.

The other hypothesis that M. Lavoisier produces to illustrate the subject is, "That the particles of caloric have a stronger mutual attraction than those of any other substance; and that these latter particles are torn asunder in consequence of this superior attraction of the particles of caloric, which forces them between the particles of other bodies, that they may be able to re-unite with each other." Now this has certainly many obstacles to encounter. If such reciprocal attractions did exist between the particles of caloric, should we not see in it some uniform, determined existence? When the mind takes a survey of the operations of the attractive power in other bodies, it is very difficult for it to conceive this superior attraction existing between the particles of caloric, at the same time observing it to be diffused throughout nature. One would think, if this superior attraction did exist, that in some one instance caloric might be found in an uncombined state. Though chemistry is a science founded on experiment, and not dependent in the least on our reasonings; though changes and combinations take place, which we never could judge of a priori; yet the reasoning from appearances should be attended to in an hypothesis, as it is the only guide we possibly can have in search of explanation. The particles of caloric may have this superior attraction existing between them; but when we enumerate circumstances, they all seem fully to contradict the idea, and of course it ought to be exploded, till something can be produced to to authorise the supposition. Supposing this highly attractive power to exist, it is very evident that the reciprocal attraction between bodies and caloric must necessarily be *eminently* great, or else the diffusion of caloric could never take place. Here a difficulty is produced. However great the attractions may be between the particles of a body, there surely can be no doubt but that they are surmounted when the body enters into combination; therefore, the question is, What end can this supposed eminent attraction between the particles of caloric answer, when the reciprocal attraction between the combining body and caloric is in itself sufficient to suspend the particles of each? We have here created a power, without giving it its use. We might as well account in this way for every combination which takes place throughout; we might as well suppose a superior attraction existing between the particles of sulphuric acid, and that its particles are forced, in consequence of this superior attraction, between those of the bodies with which it combines, in order that they may effect a re-union — instead that the reciprocal attraction between the acid, or *the elements of the acid*, and the bodies subject to such combination, is superior to the power of cohesion existing between the particles of each.

From what has been said, it would appear that the reasoning from appearances and analogy does not co-operate with the opinions, that the elasticity of gasses is either dependent *solely* on that of caloric, or that there exists that *superiority of attraction* between its particles to effect such an arrangement among the particles of bodies, from which elasticity is produced. The first opinion must be done away, I think, upon investigation; the latter seems wholly contradictory to circumstances, and bears with it that undetermined mode of explanation, that our thoughts are led away by fancy, and bewildered in a labyrinth of doubts. From such an hypothesis, no illustration can arise; it checks instead of assisting the search, and throws an obscurity over the whole. Simplicity is the first recommendation to an hypothesis; and the best mode to simplify, is to judge the darker operations of Nature by those which she performs under our more immediate observation. In this way I have endeavoured to account for the elasticity of gasses, not supposing any thing but what was authorized by natural conclusion. I have said, that there is an increased force, or rather an increase of action, given to the repulsive power existing between the particles of bodies when caloric is diffused in them; because it is evident that the particles of bodies are removed from each other when heated; and likewise I have doubted, how far caloric retains the property of elasticity* upon combination, because I see other

* If admitted to have this quality.

other bodies, upon combining, suffer change, more or less, in their properties; and likewise from the consideration that elasticity is a quality given to bodies only in particular states of existence; that it is not a peculiar quality given to any particular body, or a quality inherent in all bodies, *but common to all*, and *wholly dependant on circumstance.* Hence then it would appear that caloric, by entering into combination to a certain degree with a body, produces that change of arrangement, or that degree of separation between its particles, as to give to the repulsive power existing between them, that sufficient range of action, so far to overcome the *hinderance* of the atmospheric pressure, and *that* of the power of attraction (existing likewise between the particles) as to give it the aëriform state; and the *still* persisting efforts of this power to overcome the *resistance* of pressure, and its *re-action* upon *desisted* compression, constitute the elastic quality of the body.* Thus far we may venture to speculate; but what the change is which caloric suffers upon combination, or what degree of elasticity it gives to bodies, merely as possessing the *quality* of elasticity, we are at present ignorant of. Here then I must leave the subject to the ingenuity of others; a subject resting on speculation, without an experiment to illustrate either cause or effect. In such a situation reason has much to withstand; the ardour arising from novelty, the pleasure derived from comparison, the flights of fancy, and the natural bent the mind ever has to throw a mysterious confusion on subjects unknown, all conspire to maze it in complexity. Perhaps the still truth may never be illustrated, yet the spirit of enquiry never ought to flag: If it cannot clear up the mystery, it will ever have its good effects, in reducing the mind to a proper scale of action, in habituating it to arrange appearances in some order for discussion, and by rejecting ill-founded opinions, in guarding the more fundamental part of the science from injury.

---

### *The Progress of the Vaccine.*

WE learn from the French Official Paper, of the 5th and 8th Ventose, that the new Inoculation makes great progress in France. After naming the committee appointed to superintend

---

* Considering the yet abstruse nature of the subject, I thought it prudent not to say any thing on the action of the attractions existing between the particles, though I must say I cannot think their operations can influence much the elasticity of such bodies.

